# Supplementary material for: Conformal in-ear bioelectronics for visual and auditory brain-computer interfaces
Source: Nat Commun. 2023 Jul 14;14:4213. doi: 10.1038/s41467-023-39814-6 (PMC10349124; doi:10.1038/s41467-023-39814-6)
Supplement: Supplementary file 1 — Supplementary Information [file 41467_2023_39814_MOESM1_ESM.pdf]

Supplementary Information for

Conformal in-ear bioelectronics for visual and auditory brain-computer interfaces

Zhouheng Wang[1, 2, +], Nanlin Shi[3, +], Yingchao Zhang[2], Ning Zheng[4], Haicheng Li[1, 2], Yang Jiao[1, 2], Jiahui Cheng[1, 2], Yutong Wang[1, 2], Xiaoqing Zhang[5], Ying Chen[6], Yihao Chen[1, 2], Heling Wang[1, 2], Tao Xie[4], Yijun Wang[7], Yinji Ma[1, 2, ✉], Xiaorong Gao[3, ✉], Xue Feng[1, 2, ✉]

[1]Laboratory of Flexible Electronics Technology, Tsinghua University, Beijing, 100084, China

[2]AML, Department of Engineering Mechanics, Tsinghua University, Beijing, 100084, China

[3]Department of Biomedical Engineering, Tsinghua University, Beijing, 100084, China

[4]State Key Laboratory of Chemical Engineering, College of Chemical and Biological Engineering, Zhejiang University, Hangzhou, 310027, China

[5]Department of Otolaryngology-Head and Neck Surgery, Beijing Tongren Hospital, Capital Medical University, Beijing, 100730, China

[6]Institute of Flexible Electronics Technology of THU, Zhejiang, Jiaxing, 314000, China

[7]Institute of Semiconductors, Chinese Academy of Sciences, Beijing, 100083, China

[+]These authors contributed equally: Zhouheng Wang and Nanlin Shi.

[✉]e-mail: mayinji@tsinghua.edu.cn; gxr-dea@mail.tsinghua.edu.cn; fengxue@tsinghua.edu.cn

Supplementary Information

Table S1. Comparison of recent ear EEG electronics. 1

Figure S1. SpiralE experiences changes in its storage modulus as it goes through the stages of insertion, deformation, detection, and extraction. 2

Figure S2. The molecular structure and polymer network of the precursor monomers and the synthesized SMPs. 3

Figure S3. Characterization of the two kinds of SMPs. 4

Figure S4. Schematic illustrations of the detailed fabrication process of SpiralE. 5

Figure S5. The dynamic resistance changes of the stretchable parts of SpiralE (including all five in-ear EEG channels and electrothermal actuation layer) under 400 cycles of 10% tensile strain. 6

Figure S6. Time-temperature curves obtained during the deformation of SpiralE. Accompanying these curves are corresponding thermal images captured at various stages of the deformation process, as shown in the insets. Scale bars, 3 mm. 7

Figure S7. Skin temperature evaluation during electrothermal test. 8

Figure S8. Summary of computational studies detailing the effects of normal and shear mechanical stresses from SpiralE on human skin. 9

Figure S9. The integration between SpiralE and the external processor. 10

Figure S10. The temperature field during deformation of SpiralE in ear. Inset shows the picture of SpiralE in ear. 11

Figure S11. SpiralE-skin impedance spectroscopy of all five in-ear channels with and without gel. 12

Figure S12. The impedance diagram of all channels for one subject. 13

Table S2. The impedance of all channels for all subjects in the visual test (kilohms). 14

Figure S13. The individual spectral representations of the alpha rhythms and 10 Hz SSVEP recordings for all subjects. 15

Figure S14. Schematic diagram of the 9-target SSVEP process. 16

Figure S15. The spectral histograms of all subjects in the 9-target SSVEP test, comparing the occipital and in-ear channels. The data points (*n* = 9) of both in-ear and occipital lobe sensors represent the SNR of each stimulation class. Error bars reflect the 95% confidence intervals of these samples. 17

Figure S16. The individual classification accuracies of all subjects in the 9-target SSVEP experiment. The dashed and solid lines present the individual and group-level results of the 9 subjects, respectively. The error bands represent the 95% confidence intervals obtained from leave-one-out cross validation (*n* =12) conducted on each individual subject. 18

Figure S17. The individual classification accuracies of all subjects with SpiralE, scalp electrodes at the temporal region, and whole-scalp electrodes (*n* = 23, 24, 24, 25, and 24 for **a-e**, respectively. Statistic two-sided one sample *t*-test. Boxplot with 25-75th percentiles, mean, median line and whiskers of inner fences.). 19

References 20

Table S1. Comparison of recent ear EEG electronics.

		Ref[1]	Ref[2]	Ref[3]	Ref[4]	Ref[5]	Ref[6]	Ref[7]	Ref[8]	Ref[9]	This study
Electrodes Properties	Location	In-ear	In-ear	Auricle, mastoid	In-ear	Behind ear	Auricle, in-ear	Auricle, in-ear	Auricle, in-ear	Auricle, in-ear	In-ear
	Support	Earplug	Earphone	-	Earplug	Cap	Earmould silicone	Earmould silicone	Resin (PC/PI)	Resin (PC/PI)	SMPs
	Contact Area	Whole canal	Whole canal	-	Whole canal	Full head	Whole canal	Whole canal	Whole canal	Whole canal	Spiral
	Electrode Area	20 mm^2	-	-	40 mm^2	-	-	5.3 mm^2	60 mm^2	60 mm^2	0.785 mm^2
	Electrode Material	Ag	CNT/ PDMS	Au	Wet Ag coated nylon	Ag/AgCl	Ti/IrO_2	IrO_2	Ag	Ag	Au
	Skin Impedance (50 Hz)	-	~150 k	-	10 k	-	435 k	-	392 k	190 k	111 k
	In-ear sensors (each ear)	2	1	-	2	-	3	2	4	4	5
Features	Style	Generic	Generic	Generic	Generic	-	Customized	Customized	Generic	Generic	Generic
	Communication	Yes	No	Yes	No	-	No	Yes	Yes	Yes	Yes
	Structure	Hollow	Hollow	-	Stuffed	-	Stuffed (with a small hole)	Hollow	Hollow	Hollow	Hollow
Experiments	Visual	SSVEP/ VEP	SSVEP	2-target SSVEP<br>36-target P300: 12 bpm	-	12-target SSVEP: 30.21 ± 10.61 bpm	SSVEP	-	-	-	9-target SSVEP<br>40-target speller: 36.86 ± 15.53 bpm
	Auditory	ASSR/ AEP	ASSR/ AEP	-	ASSR	-	ASSR	ASSR	ASSR	-	AAD: Accuracy : 72.42%
	Other	-	Alpha rhythm	Alpha rhythm	-	-	Alpha rhythm	-	Alpha rhythm	Drowniness detection	Alpha rhythm
	Subjects	14	6	3	5	16	12	10	3	5	14

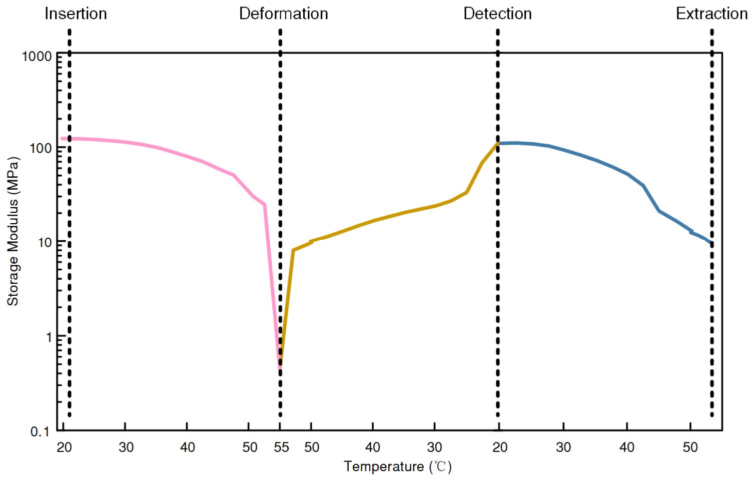

Figure S1. SpiralE experiences changes in its storage modulus as it goes through the stages of insertion, deformation, detection, and extraction.

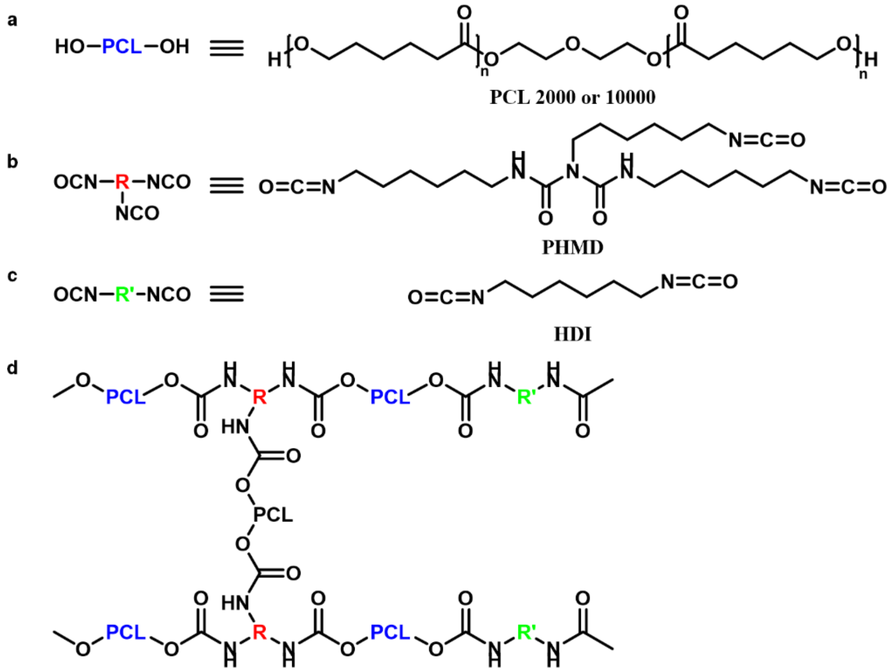

Figure S2. The molecular structure and polymer network of the precursor monomers and the synthesized SMPs. **a,** PCL. **b,** PHMD. **c,** HDI. **d,** The synthesized SMPs.

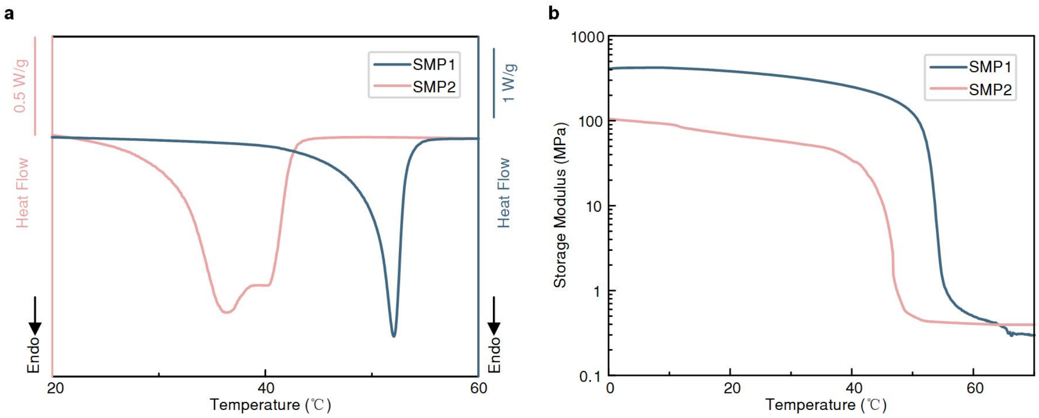

Figure S3. Characterization of the two kinds of SMPs. **a,** DSC curves. **b,** DMA curves.

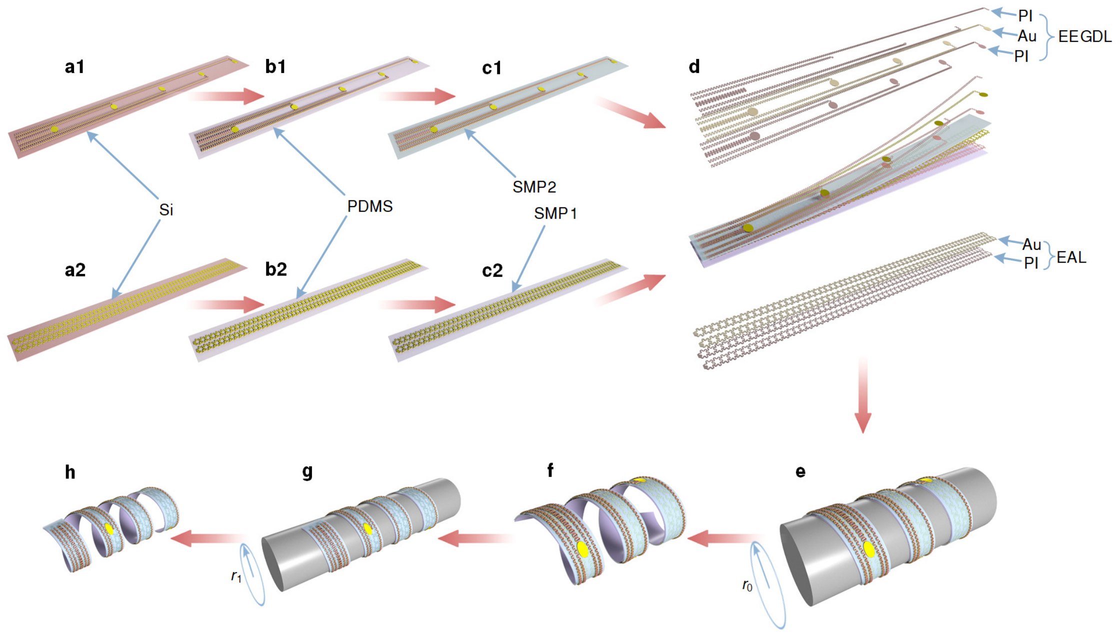

Figure S4. Schematic illustrations of the detailed fabrication process of SpiralE. **a1–a2,** Designs of the EEGDL and EAL on the Si substrates. **b1-b2,** The above layers are transferred to PDMS substrates and PI films are patterned by reactive ion etching. **c1-c2,** The two layers are transferred to the SMPs. **d,** The overall integration of SpiralE. **e-f,** Reconfiguration of the permanent shape to the designed large spiral shape with a radius of r_0. **g-h,** Change in the temporary shape of SpiralE to a small spiral configuration with a radius of r_1.

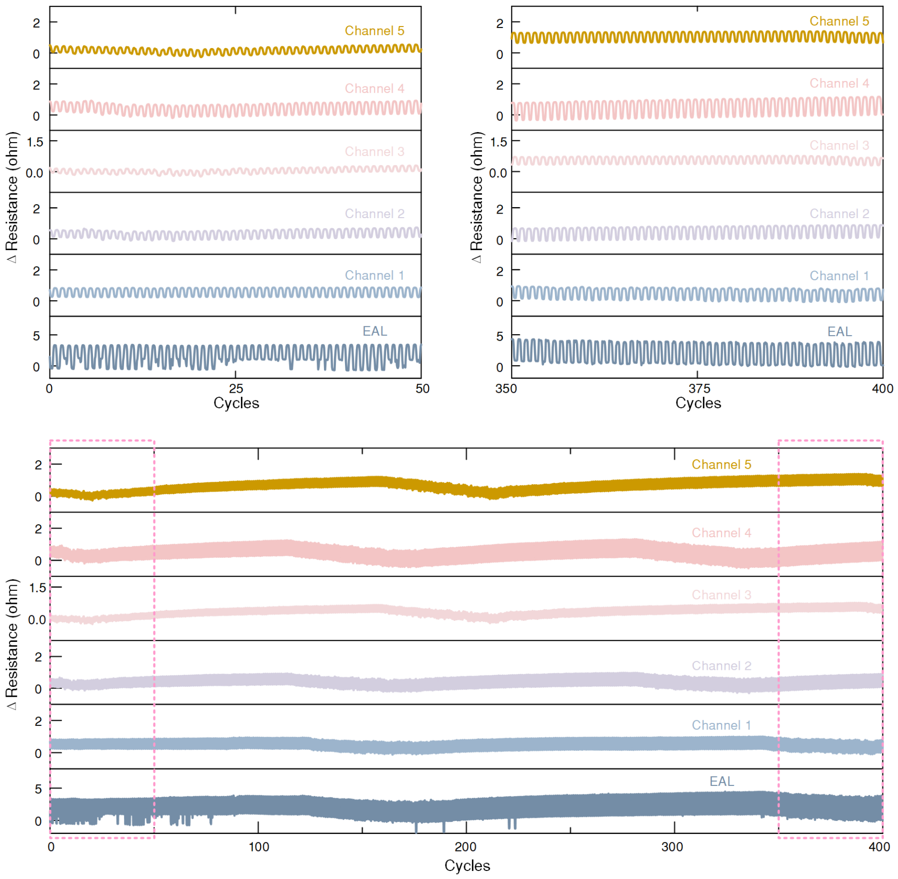

Figure S5. The dynamic resistance changes of the stretchable parts of SpiralE (including all five in-ear EEG channels and electrothermal actuation layer) under 400 cycles of 10% tensile strain.

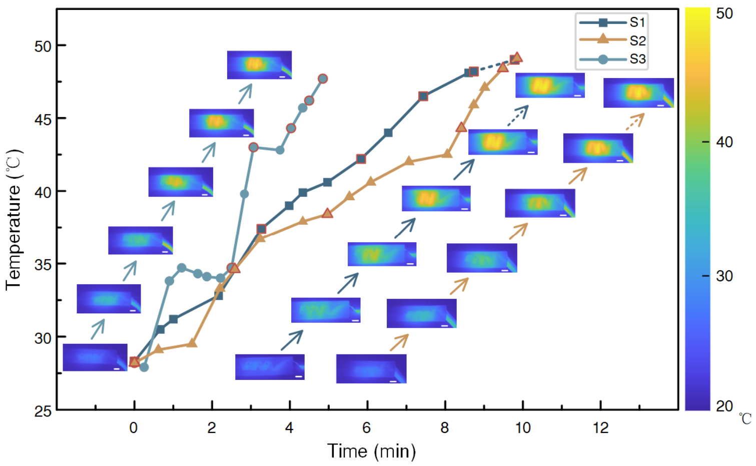

Figure S6. Time-temperature curves obtained during the deformation of SpiralE. Accompanying these curves are corresponding thermal images captured at various stages of the deformation process, as shown in the insets. Scale bars, 3 mm.

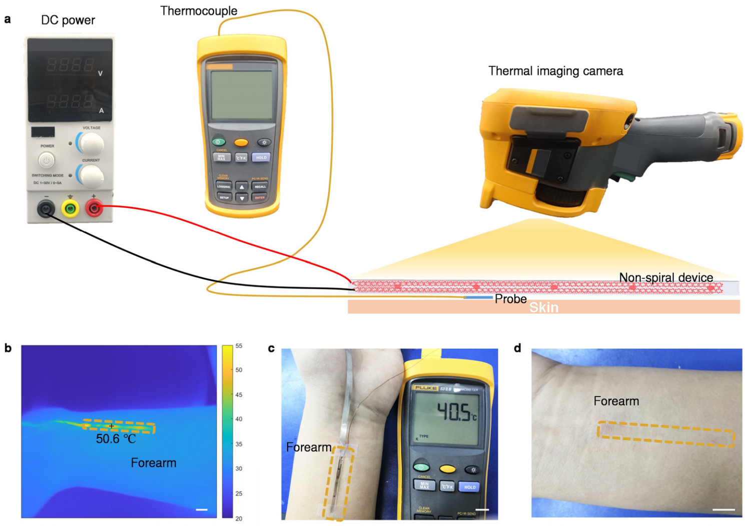

Figure S7. Skin temperature evaluation during electrothermal test. **a,** Schematic diagram of the testing process. **b,** Thermal image of the device. **c,** Skin temperature test. **d,** Peeling off the device after 3 minutes. Scale bars, 10 mm.

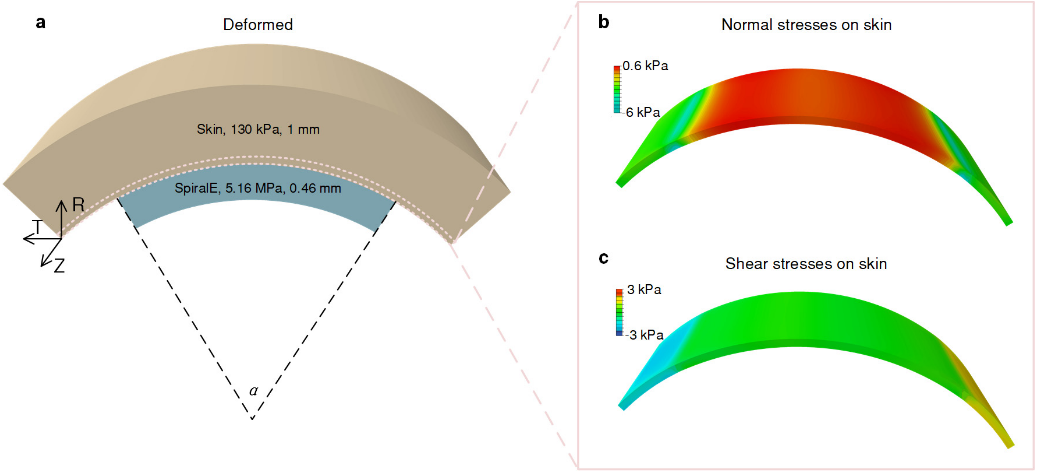

Figure S8. Summary of computational studies detailing the effects of normal and shear mechanical stresses from SpiralE on human skin. **a,** Side profile view of SpiralE attached to skin surface under bending deformation (*α*=pi/3). **b** and **c,** Finite element simulation results for a device applying normal and shear stresses on skin during bending deformations.

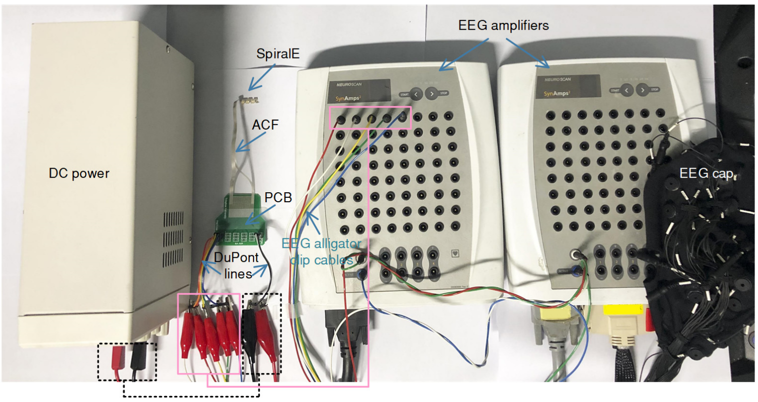

Figure S9. The integration between SpiralE and the external processor.

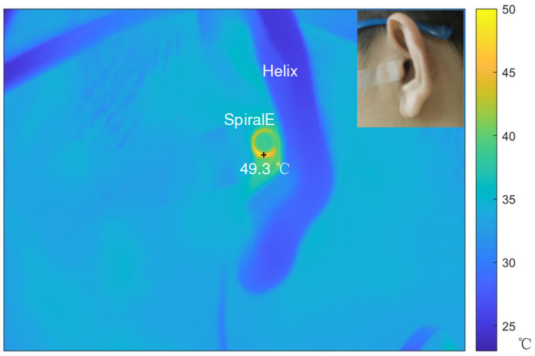

Figure S10. The temperature field during deformation of SpiralE in ear. Inset shows the picture of SpiralE in ear.

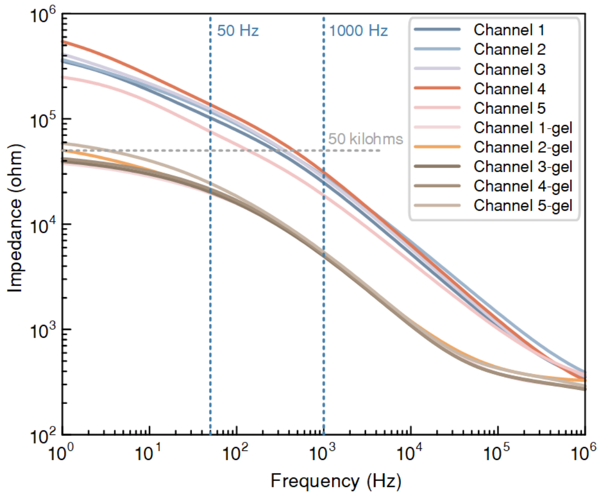

Figure S11. SpiralE-skin impedance spectroscopy of all five in-ear channels with and without gel.

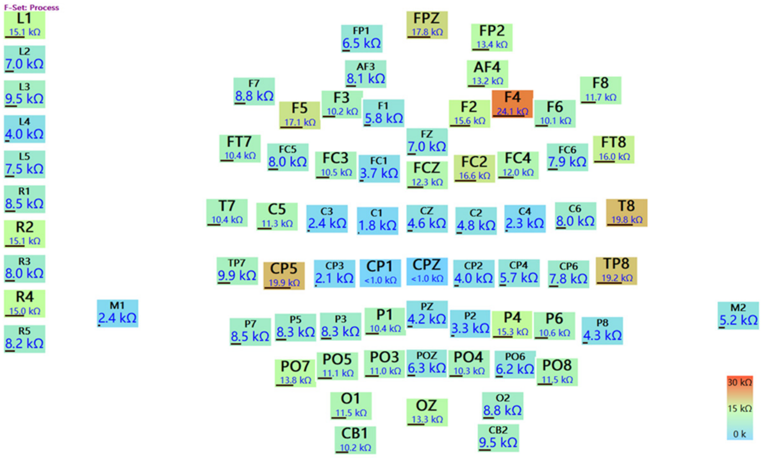

Figure S12. The impedance diagram of all channels for one subject.

Table S2. The impedance of all channels for all subjects in the visual test (kilohms).

	In-ear											Mastoid		Occipital								
	**L1**	**L2**	**L3**	**L4**	**L5**	**R1**	**R2**	**R3**	**R4**	**R5**	**Channels availability**	**M1**	**M2**	**PZ**	**PO5**	**PO3**	**POZ**	**PO4**	**PO6**	**O1**	**OZ**	**O2**
**S1**	65.7	21.9	76.5	48.9	41.2	42.8	-	36.1	37.2	35.5	7/10	2.2	3.2	3.5	9.1	9.8	4.3	11.4	4.8	9.9	8.3	11.4
**S2**	38.2	30.8	44.3	43.2	50.3	76.6	82.2	87.1	73.3	113.1	4/10	<1	1.4	10.3	4.3	2.6	11.9	11.5	6.5	8.1	10.4	9.8
**S3**	44.0	33.5	27.4	36.3	15.7	40.4	39.5	110.0	42.7	25.1	9/10	5.2	<1	4.6	12.5	6.7	9.3	6.1	10.0	2.2	6.0	5.0
**S4**	12.8	9.9	13.0	12.5	13.2	46.8	26.0	17.5	21.4	40.4	10/10	<1	4.9	1.2	7.2	7.5	3.5	<1	11.1	2.2	4.2	5.1
**S5**	6.2	6.7	9.0	20.8	7.4	3.6	3.6	4.1	4.1	3.9	10/10	5.6	11.8	1.1	1.4	1.4	1.9	1.7	2.7	1.2	1.6	1.7
**S6**	12.1	32.6	12.6	24.3	45.9	7.0	6.1	44.6	50.8	70.8	8/10	<1	4.9	3.4	6.0	2.2	3.3	4. 5	7.3	3.2	6.4	2.9
**S7**	15.7	20.6	7.6	9.5	49.7	29.1	11	15.2	9.4	65.2	9/10	<1	<1	9.5	21.8	21.8	14.7	20.4	20.6	22.0	13.1	26.7
**S8**	11.9	5.6	8.6	7. 7	<1	41.7	40.9	14.4	17.0	25.8	10/10	3.7	3.6	11.0	8.8	7.9	3.1	12.2	3.6	12.9	10.3	7.1
**S9**	15.3	7.6	9.5	4.0	7.32	7.9	14.4	7.4	13.6	7.5	10/10	2.5	5.3	4.2	10.0	10.3	6.0	9	5.7	10.9	12.4	8.0

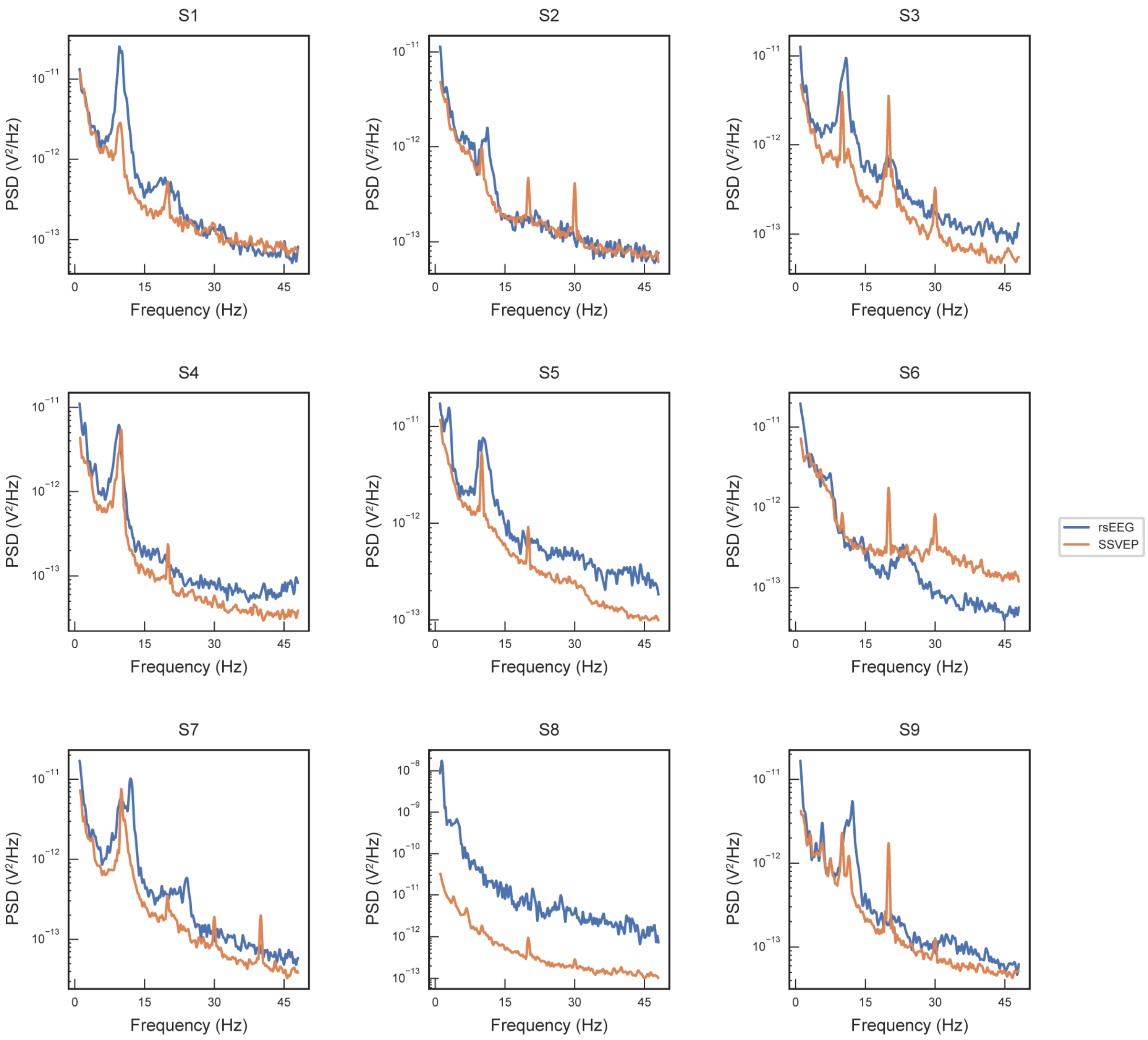

Figure S13. The individual spectral representations of the alpha rhythms and 10 Hz SSVEP recordings for all subjects.

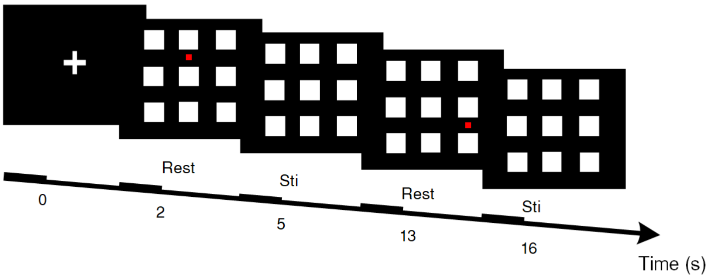

Figure S14. Schematic diagram of the 9-target SSVEP process.

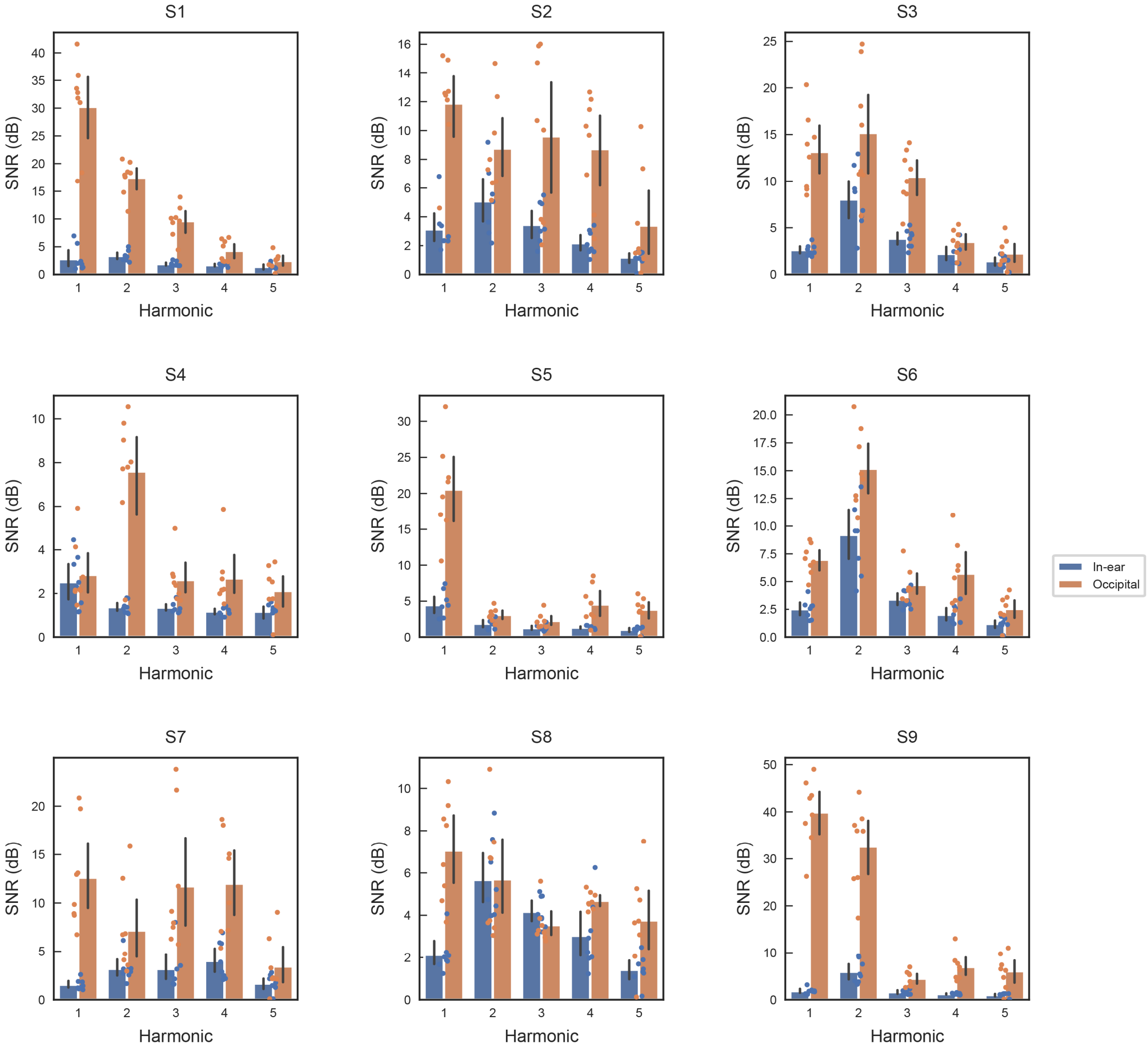

Figure S15. The spectral histograms of all subjects in the 9-target SSVEP test, comparing the occipital and in-ear channels. The data points (n = 9) of both in-ear and occipital lobe sensors represent the SNR of each stimulation class. Error bars reflect the 95% confidence intervals of these samples.

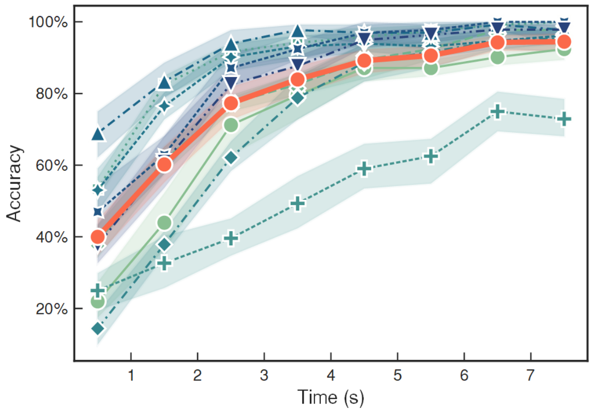

Figure S16. The individual classification accuracies of all subjects in the 9-target SSVEP experiment. The dashed and solid lines present the individual and group-level results of the 9 subjects, respectively. The error bands represent the 95% confidence intervals obtained from leave-one-out cross validation (*n* =12) conducted on each individual subject.

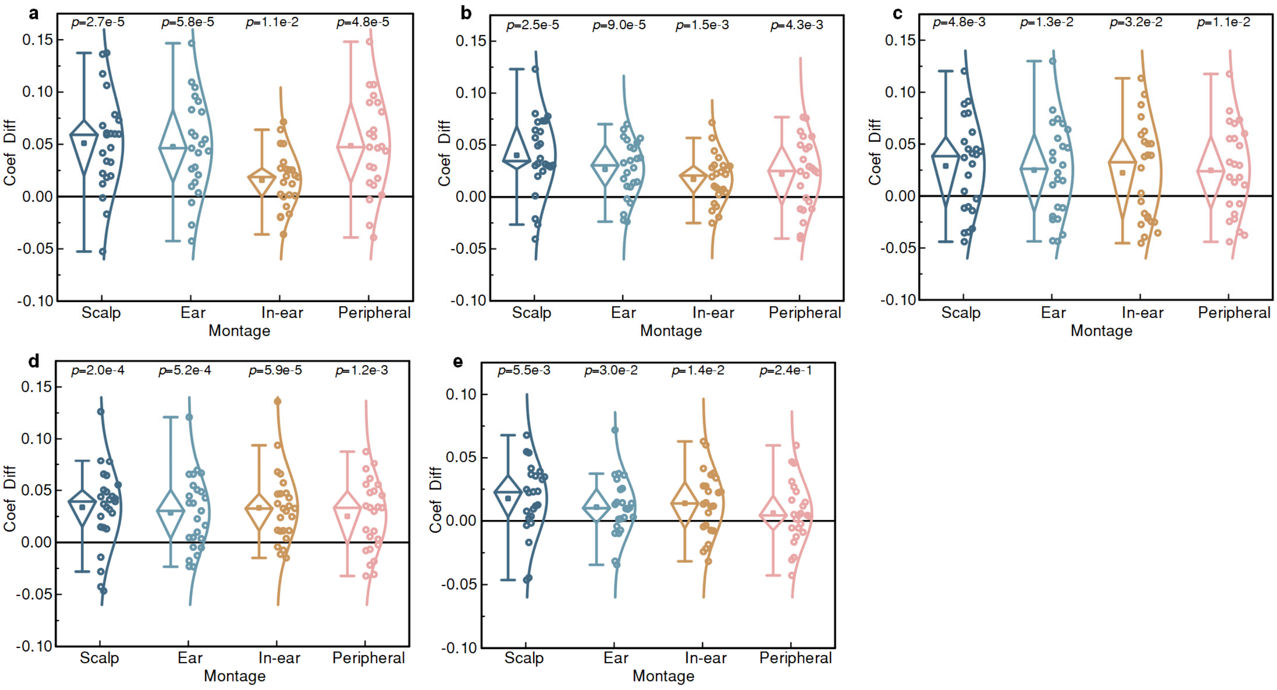

Figure S17. The individual classification accuracies of all subjects with SpiralE, scalp electrodes at the temporal region, and whole-scalp electrodes (n = 23, 24, 24, 25, and 24 for **a-e**, respectively. Statistic two-sided one sample t-test. Boxplot with 25-75th percentiles, mean, median line and whiskers of inner fences.).

References

1 Kidmose, P., Looney, D., Ungstrup, M., Rank, M. L. & Mandic, D. P. A study of evoked potentials from ear-EEG. *IEEE Trans. Biomed. Eng.* **60**, 2824-2830, (2013).

2 Hoon Lee, J. *et al.* CNT/PDMS-based canal-typed ear electrodes for inconspicuous EEG recording. *J. Neural Eng.* **11**, 046014, (2014).

3 Norton, J. J. S. *et al.* Soft, curved electrode systems capable of integration on the auricle as a persistent brain-computer interface. *Proc. Natl. Acad. Sci. U. S. A.* **112**, 3920-3925, (2015).

4 Goverdovsky, V., Looney, D., Kidmose, P. & Mandic, D. P. In-Ear EEG from viscoelastic generic earpieces: robust and unobtrusive 24/7 monitoring. *IEEE Sens. J.* **16**, 271-277, (2016).

5 Wang, Y. T. *et al.* An online brain-computer interface based on SSVEPs measured from non-hair-bearing areas. *IEEE Trans. Neural Syst. Rehabil. Eng.* **25**, 14-21, (2017).

6 Kappel, S. L., Rank, M. L., Toft, H. O., Andersen, M. & Kidmose, P. Dry-contact electrode ear-EEG. *IEEE Trans. Biomed. Eng.* **66**, 150-158, (2019).

7 Bertelsen, A. R. *et al.* Generic dry-contact ear-EEG. // *2019 41st Annual International Conference of the IEEE Engineering in Medicine and Biology Society (EMBC)*, 5552-5555, (2019).

8 Kaveh, R. *et al.* Wireless user-generic ear EEG. *IEEE Trans. Biomed. Circuits Syst.* **14**, 727-737, (2020).

9 Schwendeman, C., Kaveh, R. & Muller, R. Drowsiness detection with wireless, user-generic, dry electrode ear EEG. // *2022 44th Annual International Conference of the IEEE Engineering in Medicine & Biology Society (EMBC)*, 9-12, (2022).
