# Supplementary material for: Conformal in-ear bioelectronics for visual and auditory brain-computer interfaces
Source: Nat Commun. 2023 Jul 14;14:4213. doi: 10.1038/s41467-023-39814-6 (PMC10349124; doi:10.1038/s41467-023-39814-6)
Supplement: Supplementary file 3 — Description of Additional Supplementary Files [file 41467_2023_39814_MOESM3_ESM.pdf]

<u>**Description of Additional Supplementary Files**</u>

Supplementary Movie 1: Further deformation of SpiralE under electrothermal actuation after removal from ear.

Supplementary Movie 2: Shape recovery of SpiralE in 50 °C water.

Supplementary Movie 3: Deformation of SpiralE under electrothermal actuation in a tube.

Supplementary Movie 4: SpiralE expands and deforms at complex curvature radii in a right-angle elbow.

Supplementary Movie 5: SpiralE adaptively conforms to a tube with nonuniform curvatures.

Supplementary Movie 6: One subject uses SpiralE in a calibration-free 40-target online SSVEP speller experiment and types target phrases successfully.
