# Supplementary material for: Conformal in-ear bioelectronics for visual and auditory brain-computer interfaces
Source: Nat Commun. 2023 Jul 14;14:4213. doi: 10.1038/s41467-023-39814-6 (PMC10349124; doi:10.1038/s41467-023-39814-6)
Supplement: Supplementary file 10 — Reporting Summary [file 41467_2023_39814_MOESM10_ESM.pdf]

nature portfolio

Corresponding author(s): Yinji Ma, Xiaorong Gao, and Xue Feng

Last updated by author(s): Jun 15, 2023

Reporting Summary

Nature Portfolio wishes to improve the reproducibility of the work that we publish. This form provides structure for consistency and transparency in reporting. For further information on Nature Portfolio policies, see our Editorial Policies and the Editorial Policy Checklist.

Statistics

For all statistical analyses, confirm that the following items are present in the figure legend, table legend, main text, or Methods section.

n/a	Confirmed	
☐	☒	The exact sample size (*n*) for each experimental group/condition, given as a discrete number and unit of measurement
☐	☒	A statement on whether measurements were taken from distinct samples or whether the same sample was measured repeatedly
☐	☒	The statistical test(s) used AND whether they are one- or two-sided<br>*Only common tests should be described solely by name; describe more complex techniques in the Methods section.*
☒	☐	A description of all covariates tested
☐	☒	A description of any assumptions or corrections, such as tests of normality and adjustment for multiple comparisons
☐	☒	A full description of the statistical parameters including central tendency (e.g. means) or other basic estimates (e.g. regression coefficient) AND variation (e.g. standard deviation) or associated estimates of uncertainty (e.g. confidence intervals)
☐	☒	For null hypothesis testing, the test statistic (e.g. *F*, *t*, *r*) with confidence intervals, effect sizes, degrees of freedom and *P* value noted<br>*Give P values as exact values whenever suitable.*
☒	☐	For Bayesian analysis, information on the choice of priors and Markov chain Monte Carlo settings
☒	☐	For hierarchical and complex designs, identification of the appropriate level for tests and full reporting of outcomes
☐	☒	Estimates of effect sizes (e.g. Cohen's *d*, Pearson's *r*), indicating how they were calculated

Our web collection on statistics for biologists contains articles on many of the points above.

Software and code

Policy information about availability of computer code

Data collection	Curry 8 (NeuroScan. Inc)<br>Impedance Analysis Interface (PSM 1700, Newtons4th Ltd)
Data analysis	Python 3.8, Origin 2022, eelbrain 0.39, autoreject 0.4.2. The custom codes developed in this study have been stored in Zenodo (https://doi.org/10.5281/zenodo.7748035).

For manuscripts utilizing custom algorithms or software that are central to the research but not yet described in published literature, software must be made available to editors and reviewers. We strongly encourage code deposition in a community repository (e.g. GitHub). See the Nature Portfolio guidelines for submitting code & software for further information.

Data

Policy information about availability of data

All manuscripts must include a data availability statement. This statement should provide the following information, where applicable:

- Accession codes, unique identifiers, or web links for publicly available datasets
- A description of any restrictions on data availability
- For clinical datasets or third party data, please ensure that the statement adheres to our policy

The collected visual and auditory experiments are provided via Zenodo (https://doi.org/10.5281/zenodo.7748035). A detailed manual for the data analysis and

deployment can be found in the Zenodo repository. Raw EEG data can be obtained from corresponding authors on reasonable request. Source Data are provided with this paper.

Human research participants

Policy information about studies involving human research participants and Sex and Gender in Research.

Reporting on sex and gender	Sex was considered in the study design, and the study included 11 males and 3 females. Participants were solicited to self-report their biological sex. Prior to the experiment, a comprehensive explanation of the experimental protocol was provided to all participants, and they signed the informed consents.
Population characteristics	Nine subjects (7 males and 2 females) with normal vision and an average age of 26.89 years (s.d. 3.1) participated in the visual experiments, and 5 subjects (4 males and 1 female) with normal hearing and an average age of 25 years (s.d. 1.7) participated in the auditory experiments.
Recruitment	Participants were enrolled in campus and community. Compensation were paid 100 RMB/hour. The BCI performance may be affected by inter-subject variability, which could introduce potential biases to the reported results.
Ethics oversight	All measurements were approved by the Institution Review Board of Tsinghua University (20220023).

Note that full information on the approval of the study protocol must also be provided in the manuscript.

Field-specific reporting

Please select the one below that is the best fit for your research. If you are not sure, read the appropriate sections before making your selection.

☒ Life sciences ☐ Behavioural & social sciences ☐ Ecological, evolutionary & environmental sciences

For a reference copy of the document with all sections, see nature.com/documents/nr-reporting-summary-flat.pdf

Life sciences study design

All studies must disclose on these points even when the disclosure is negative.

Sample size	Fourteen participants (11 males and 3 females) were recruited for this study without a formal sample size calculation. This sample size falls within the typical enrollment in BCI studies, which usually ranges from 10 to 20 participants. Thus, it is considered sufficient based on relevant research.
Data exclusions	No data were excluded.
Replication	The experiments in our study were replicated with different participants. Each subject participated in the same experimental paradigm, allowing for replication across multiple groups. The results exhibited high similarity, indicating good reproducibility across various measurements conducted in this study.
Randomization	Participants were chosen randomly from a mixed-gender sample, with an average age of 26 years. However, comparative group performance is not relevant to the research purpose.
Blinding	All participants were informed about the experimental protocol but remained unaware of the test results, except for the subject in Supplementary Movie 6, who witnessed the target phrases being typed on the screen during the experiment. None of the participants were involved in data processing.

Reporting for specific materials, systems and methods

We require information from authors about some types of materials, experimental systems and methods used in many studies. Here, indicate whether each material, system or method listed is relevant to your study. If you are not sure if a list item applies to your research, read the appropriate section before selecting a response.

Materials & experimental systems

n/a	Involved in the study
☒	☐ Antibodies
☒	☐ Eukaryotic cell lines
☒	☐ Palaeontology and archaeology
☒	☐ Animals and other organisms
☒	☐ Clinical data
☒	☐ Dual use research of concern

Methods

n/a	Involved in the study
☒	☐ ChIP-seq
☒	☐ Flow cytometry
☒	☐ MRI-based neuroimaging
